# Part of the Game? Exploring the Prevalence and Normalization of Gambling in Belgian Sports Clubs

**DOI:** 10.3390/ijerph19116527

**Published:** 2022-05-27

**Authors:** Bram Constandt, Johan Rosiers, Jolien Moernaut, Stef Van Der Hoeven, Annick Willem

**Affiliations:** 1 Department of Movement and Sports Sciences, Ghent University, Watersportlaan 2, 9000 Ghent, Belgium; stef.vanderhoeven@ugent.be (S.V.D.H.); annick.willem@ugent.be (A.W.); 2 The Flemish Centre of Expertise on Alcohol and Other Drugs, Vanderlindenstraat 15, 1030 Brussels, Belgium; johan.rosiers@vad.be (J.R.); jolien.moernaut@vad.be (J.M.)

**Keywords:** gamblification of sports, gambling, gambling education, gambling harm, normalization, problem gambling, sports betting, sports clubs, sports ethics, sports integrity

## Abstract

Gambling and sports are entangled in a close relationship. However, little remains known about gambling behaviors and perceptions in sports. Drawing on normalization theory, this study explores the prevalence and predictors of problem gambling as well as the normalization of gambling (including its availability and accessibility, prevalence, and socio-cultural accommodation) in sports clubs. A cross-sectional study design was implemented, based on an online survey completed by 817 Belgian sports club actors. This survey consisted of the Problem Gambling Severity Index (PGSI) and questions about personal and socio-cultural factors regarding gambling. Data were analyzed with SPSS 26 software, using descriptive statistics and an ordinal logistic regression analysis. These analyses exposed being male, being aged 26–35 years old, and being involved in football (soccer) as factors that might be linked with higher levels of problem gambling in sports. Furthermore, sports betting is especially shown to be normalized in sports clubs given its prevalence, and its frequently organized and discussed character. Moreover, respondents disclosed a lack of formal rules (96%) and education initiatives (98.7%) on gambling in their sports club. Given the indicated support for gambling regulations and educational measures, this study may inform sports organizations about how to help denormalize gambling.

## 1. Introduction

Sports and gambling are entangled in an ambiguous relationship [1]. On the one hand, it is sometimes argued that gambling on sports represents an indispensable cultural aspect of consuming sports [2,3]. Moreover, in times of ever-increasing commercialization, many sports organizations rely heavily on the generous sponsoring of gambling companies [4,5,6]. On the other hand, the associated harms of (problem) gambling (for example, relationship breakdowns, debts, anxiety, depression, and suicide), numerous incidents of gambling-related match-fixing, and the increase in aggressive gambling advertisement mark a potential turning point concerning society’s perspective on gambling [7,8]. More precisely, a public and political debate on the omnipresence and legitimization of gambling in society as well as on the “gamblification” of sports is currently ongoing in different countries, such as the UK, Belgium, Spain, and Italy [9,10,11,12,13].

Scholarly work on the consequences of the close relationship between gambling and sports has not been coupled with many insights into the prevalence of gambling in sports settings [14,15]. Despite recent work showcasing elite athletes as a group that is at risk for problem gambling, little remains known about gambling behaviors and perceptions of other actors in sports [14,15,16]. Applying a socio-cultural perspective (see for example, [2,17]), this study examines the gambling behaviors of Belgian sports clubs actors (for example, players, coaches, board members, and volunteers). The twofold objective of this study is (a) to deepen our knowledge about the prevalence and potential predictors of problem gambling in these actors, and (b) to explore the extent of gambling normalization in sports clubs. The lens of normalization theory—underexplored in sports management—is thereby used. Pointing to a certain paradox, this study sheds light on how the socio-cultural context within sports clubs can contribute to both gambling normalization (by means of the creation, accommodation, and institutionalization of positive socio-cultural norms) and denormalization (by means of education and regulation). Regulation (formal rules) and educational initiatives are key to raise awareness in sports club actors, to tackle gambling harms, and to denormalize gambling [14,15]. Hence, this study adds to the scientific body of literature on gambling and the role of sports organizations, by highlighting socialization mechanisms in sports clubs that legitimize gambling [18,19]. 

## 2. Literature Review

### 2.1. (Problem) Gambling in Sports

As indicated above, much scholarship has already emphasized the close relationship between sports on the one hand and gambling (in general) and sports betting (in particular) on the other hand. Sports as an institution has thereby been shown to be an ideal vehicle to promote sports betting products, for instance through advertising and marketing during sports events, and (shirt) sponsorship in sports organizations [5,6,9,13,16,20]. Some academics point to potential positive outcomes of recreational gambling on stress levels and life satisfaction [21,22]. However, public health scholars suggest that the “gamblificiation” of sports contributes to a rise in problem gambling (gambling more frequently and/or for more money than intended) and associated harms (for example, financial, relational, and job-related issues) [11,16,22]. Research also shows that individuals experiencing gambling disorder account for a considerable share of gambling companies’ revenues, thereby indicating a societal cost [23].

Research on the consequences of sports betting has been accompanied by studies that aim to determine the profile(s) of sports bettors. Guided by the question “who bets on sports?”, scholars have shed light on the characteristics of sports bettors in Germany [24]. The results of their study highlight “that the typical sports-bettor is 32 years old and male, has a low household income, is highly interested in sports, and is willing to take risks” [24] (p. 391). Australian research on the profile of those who place bets on micro sports events points to similar results concerning the influence of risk tendency and age, but also emphasizes the link with being single and well educated [25]. A strong interest in consuming sports is a recurring factor in most studies on this topic. At the same time, little is still known about the potential role of an active involvement in sports (for example, playing or managing sports) on being engaged in sports betting and other gambling products [14]. German survey research suggests that the typical sports bettor is little actively engaged in sports, but more (recent) insights in this regard are amply needed [26].

Existing studies on gambling by sports actors have mainly focused on gambling behaviors of elite athletes, indicating that these athletes show significantly more problem gambling compared to the general population [4,27]. Their relatively young age, a self-perceived strong knowledge of sports, a tendency towards risky behavior, occupational uncertainty, a lot of free time, and—in some cases—abundant financial abilities are highlighted as some of the susceptibility conditions for elite athletes [4,27,28,29]. Nevertheless, scarce knowledge remains present about the background of gambling behaviors of non-elite (amateur) athletes and other important actors in sports clubs, such as coaches and board members [14,15,27]. The gambling behaviors of non-elite sports actors—who are often engaged in sports as volunteers enjoying their hobby—are not necessarily identical to those of elite athletes. Hence, the first twofold research question focuses on problem gambling in sports club actors as follows:

**RQ** **1a**. *What is the prevalence of problem gambling in sports club actors?*

**RQ** **1b**. *What are the predictors of problem gambling in sports club actors?*

An enhanced understanding about the prevalence and predictors of problem gambling in sports would support the determination of the profiles of individuals experiencing gambling disorder in sports. Such knowledge is required to inform and guide initiatives to prevent gambling-related harms [11,30]. In a broader sense, such knowledge would also help to evaluate the extent in which different sorts of gambling products (for example, casino games, lotteries, sports betting) are present and socially accepted in sports [18]. Referring to this presence and social acceptance of gambling, scholars have used normalization theory to frame gambling as a normalized issue in sports and society [17,19].

### 2.2. The Normalization of Gambling

Normalization theory is rooted in the work of the French philosopher Michel Foucault on how societies use institutions, power, and dominant discourses to legitimize and normalize how certain people that are deemed abnormal (for example, homosexuals and prisoners) are treated [31,32,33,34]. Over the years, normalization theory has been extensively applied in numerous studies to examine all kinds of social practices. Such studies aim to improve our understanding about how these social practices have “obtained a firm footing in society” [35] (p. 943). More precisely, normalization theory helps to understand how social practices become rationalized, socialized, routinized, and institutionalized, yet in a taken for granted and often unconscious manner [35]. Gambling is contemplated as such a normalized social practice that is generally seen as fitting in today’s society [11,27,36]. Drawing on normalization theory also helps to provide additional, context-embedded understandings about gambling beyond the insights offered by mere psychological, biological, or rational choice theories that focus on individual decisions and responsibilities only [19,33,37].

Supported by increasing liberalization and deregulation trends over the past three decades, gambling has turned into a fast growing and economically very profitable business [11,38]. The size of the global gambling market reached nearly $449.3 billion in 2018, with a pre-COVID-19 estimated annual growth rate of nearly 6% for the coming years [39]. Simultaneously with the growth of the gambling industry and its visibility, gambling has turned into a socially broadly accepted and regularly implemented leisure activity and cultural lifestyle [2,11,27,38]. Enabled by dominant discourses on “responsible gambling”, these positive social norms towards gambling stimulate gambling intentions and behaviors [5,33]. Yet, the health and social risks of gambling are often inadequately highlighted by gambling companies and authorities, leading to insufficient awareness in gamblers [18,33,40].

Drawing on empirical research in Australia, Thomas et al. [19] have explained that several indications of the normalization of gambling are currently present in society. For example, gambling has become broadly accessible and available. Gamblers used to have to visit specific venues (such as casinos and bookmakers) to gamble. However, since the rise of online gambling, they can now literally gamble from everywhere around the globe. High trying rates of different gambling products (both online and offline) are present in society, while gambling is also recently and regularly implemented by a considerable proportion of the population. Additionally, gambling is both socially and culturally accommodated by—among other aspects—gambling advertising and its cultural alignment with other activities (for example, sports) [11,19]. In this light, Thomas and colleagues [19] (p. 6) have defined the normalization of gambling as follows:

“The interplay of socio-cultural, environmental, commercial, and political processes which influence how different gambling activities and products are made available and accessible, encourage recent and regular use, and become an accepted part of everyday life for individuals, their families, and communities.”

Discussing the normalization of gambling in New Zealand, Mack [41] (p. 1) has further suggested to frame gambling as a so-called “ausugenic environment”; an environment in which gambling is “embedded in the cultural attitudes and behavior of a society to the extent that it is no longer considered to be an abnormal or noteworthy activity”. Next to the influence of gambling marketing, Deans et al. [18] identified three main drivers of the normalization of sports betting among young men in Australia, being (a) a general social acceptance (and overestimation of the prevalence) of gambling, (b) prevalent peer discussions on gambling that create an identity and sense of belonging, and (c) the existence of dominant social norms in favor of gambling that may lead to social pressure. Studying these gambling normalization drivers in a university student sample in Canada, Sanscartier et al. [42] have shown that positive norms towards gambling are often coupled with positive norms towards other addictive behaviors such as alcohol.

Whereas a considerable amount of research attention has thus already been dedicated to the study of the normalization of gambling in society—especially in Australia, New Zealand, Canada, and the UK—little remains known about the normalization of gambling in sports organizations and settings in a European context [15]. This is unfortunate given the close relationship between sports and gambling, as well as the strong societal impact of sports. Vinberg et al. [15] have indicated that sports settings may directly stimulate gambling behaviors as gambling is a popular topic of conversation in locker rooms. Furthermore, gambling success is believed to function as an additional measure of performance in the highly competitive and results-oriented sports world (striving to be not only good on the playing field of sports but also with regard to the gambling table), while it also offers thrill and reward to many sports actors [15].

Following general calls to examine the underlying socio-cultural mechanisms of the normalization of gambling [18,19] and specific calls to better understand the role of sports environments [14,15], this study aims to explore the role of sports clubs. Being one of the cornerstones of organized sports in Europe, sports clubs can operate as stimulators as well as regulators and educators in relation to gambling [14]. On the one hand, sports clubs function as influential social environments. As gambling is considered a popular pastime and much-discussed topic among especially young adults in sports [3,11,18], sports clubs could contribute to the normalization of gambling when no initiatives are implemented to critically discuss gambling or to limit gambling ads and sponsorships [16]. Moreover, many sports clubs organize or at least facilitate sports betting, poker tournaments, and other gambling activities [5]. On the other hand, sports clubs can inform their members about those gambling behaviors that are (not) allowed, gambling risks, and potential social and health-related harms by means of well-developed and broadly supported gambling policies, such as formal rules and educational initiatives [14]. As such, a second research question is formulated:

**RQ** **2**. *To what extent is gambling normalized in sports clubs?*

When assessing this second research question, specific attention is dedicated to the different indications of the normalization of gambling, as highlighted and discussed by Thomas and colleagues [19]. More precisely, the prevalence (estimation) of different gambling products (for example, lotteries, sport betting, casino games, poker, and machine slots), their availability and accessibility, and their socio-cultural accommodation in sports clubs are examined. Additionally, focus is also put on existing and desired gambling policies within the respective sports clubs of our respondents. Gaining insights in this regard might inform public authorities and sports organizations about both existing and preferred gambling policy measures.

## 3. Materials and Methods

### 3.1. Study Design, Procedure, and Sampling

This study was executed in Flanders, which is the northern, largest, and mainly Dutch speaking region of Belgium. A quantitative, cross-sectional study design was developed as a collaborative effort of the authors’ university and VAD (the Flemish Centre of Expertise on Alcohol and other Drugs). VAD is the official partner organization of the Flemish government when it comes to the prevention of harms caused by alcohol, drugs, psychoactive medication, and gambling and gaming from a public health perspective. Based on existing research, an online, anonymous survey was compiled, targeting gambling behaviors and perceptions, and individual and socio-cultural (for example, sports club) factors. After ethical clearance by an independent ethics committee of the authors’ university, participation of as many official sports federations as possible was requested by in person, e-mail, and telephone contact. Subsequently, participating sports federations administered the survey to the sports clubs and their members via their internal mailing lists and social media channels. The survey was also distributed by the newsletters and social media channels of the authors’ university and VAD, and by emailing local sports officials.

The survey remained online and active from 16 December 2019 until 16 March 2020. To ensure reliable responses, survey participation was strictly confidential and anonymous, on an individual, team-related, and sports club level. Accordingly, due to the lack of information of the team or sports club individuals belong to, individual data could not be aggregated to the club or team levels and all analyses were executed on a personal level only. The survey was open to all people involved in a sports club, regardless of their type of sports, level of play, intensity of involvement, or sports club function. A minimum age of 16 years old was required to participate, but respondents of 16 and 17 years old were requested (albeit we could not control this) to obtain the approval of at least one of their parents prior to completing the survey. Although the official minimum age to participate in gambling in Belgium is 18 years old (and 21 years for casino games), previous school-based research of VAD has indicated that Belgian minors are often already clandestinely involved in a number of gambling activities.

### 3.2. Survey and Measures

The survey contained four main sections, respectively on (a) the respondents’ demographics (for example, age and gender) and involvement in sports (for example, type of sports, sports club function(s)), (b) their gambling behaviors in general and sports betting practices in particular (for example, what kind of gambling products they engaged in, how frequently, and for how much money), (c) the “Problem Gambling Severity Index” (PGSI) [43], and (d) perceptions on the present and desired socio-cultural environment regarding gambling in their club. Questions regarding respondents’ engagement in different gambling products (for example, lotteries, poker, sports betting, bingo, casino games) were based on existing gambling research [14,44] and were measured by means of Likert scales, with five answer options, ranging from “never” to “daily”.

Problem gambling was assessed by means of the PGSI, a standardized scale consisting of nine questions. Completing the PGSI, respondents self-reported on their gambling behaviors during the past 12 months, answering such questions as: “have you bet more than you could really afford to lose?”. For each question, four answer options were present (“never”, “sometimes”, “most of the time”, and “always”). These four answer options represent a score between 0 and 3. Afterwards, respondents’ total PGSI score can be calculated, enabling a distinction between five categories: non gamblers, non-problematic gamblers (a total score of 0), low-risk gamblers (a total score of 1–2), moderate risk gamblers (a total score of 3–7), and problematic gamblers (a total score of 8 or above). Ferris and Wynne [43] developed the PGSI as part of their Canadian Problem Gambling Index (CPGI). While other, more recent instruments to measure the severity of problem gambling are available, the PGSI was chosen given its internal consistency, reliability, construct validity, and continuing popularity in both research and practice [45].

Using the PGSI also enabled us to build on—and cautiously compare with—Sciensano’s [46] population health survey, in which the same instrument is used. Sciensano is an official Belgian public research institution that is responsible (among other tasks) for the administration and analysis of the quinquennial health survey. This health survey encompasses a broad range of questions about numerous health-related themes and behaviors (including gambling). The survey targets 10,000 Belgians to obtain a representative view on the health situation of the Belgian population.

Respondents’ perceptions of gambling behaviors and policies in their club were targeted via a mix of five-point Likert scale, yes/no, and open-ended questions. For example, respondents were asked about whether (and if yes, which) gambling regulations and preventive measures were present in their sports club, whether gambling was discussed with sports friends and coaching staff, what their attitudes (and those of their sports friends) towards sports betting were (for example, “is sports betting important in your life?” and “do most of your sports friends endorse participation in sports betting?”), if pressure to gamble had been experienced (for example, “do you feel pressure by your sports friends to participate in sports betting?” and “is participation in sports betting important to really belong in your team or club?”), why they engaged in sports betting (for example, “is it a way to earn money?” and “is it a way to relax?”), and whether they had gambled on their own sports matches.

### 3.3. Data Analysis

All of the data were analyzed by means of SPSS 26 software. Descriptive statistics (for example, frequencies) and an ordinal logistic regression analysis were used to assess the prevalence (*RQ 1a*) and predictors (*RQ 1b*) of problem gambling. In line with existing problem gambling research, respondents’ total PGSI score was calculated and used to make a distinction between the following five categories: non gamblers, non-problematic gamblers (a total score of 0), low-risk gamblers (a total score of 1–2), moderate risk gamblers (a total score of 3–7), and problematic gamblers (a total score of 8 or above). The five categories of the PGSI operated as the dependent variable of the ordinal logistic regression analysis. Demographics (gender and age category) and sports-related characteristics (type of sports, sports club function, and level of play) were added as independent variables to the model. When considering the type of sports, only the five most strongly represented sports in our sample in terms of the respondents’ involvement (football, tennis, volleyball, cycling, and basketball) were included to ensure that all independent variables consisted of a sufficiently large sample size. Descriptive statistics were also used to analyze the extent of gambling normalization by looking into the prevalence, availability and accessibility, and socio-cultural accommodation of different gambling products, as well as to examine existing and preferred club level gambling policies (*RQ 2*).

## 4. Results

### 4.1. Demographics

In total, 817 fully completed surveys were collected. The average age of the respondents was 35.2 years old, whereas two-thirds of them were male. Respondents could indicate different functions within their sports club. 77.8% (*n* = 636) was involved as an athlete, while board members (23.6%), coaches (18.1%), and volunteers (12.2%) were also represented. In this study, volunteers were considered people that were active in such club activities as operating the canteen and cleaning the dressing rooms. Most of the respondents (62.8%) were active on an amateur/recreational level. Respondents were involved in a number of different sports, including (but not limited to) football (26.6%), tennis (12.1%), volleyball (9.7%), cycling (6.9%), and basketball (5.8%). A more detailed overview of the sociodemographic constitution of the study’s sample can be found in Table 1.

### 4.2. The Prevalence and Predictors of Problem Gambling (RQ 1)

Approximately one-third of our sample (30.1%) had taken part in any kind of gambling activity involving a monetary stake during the 12 months prior to completing the survey. As illustrated in Table 2, last-year gamblers (those who had gambled in the past 12 months) disclosed their engagement in a wide range of different gambling activities, with a varying regularity between once a year and more than once a week. In terms of problem gambling, our total sample showed low risk (5.7%), moderate risk (4.2%) and more problematic (0.8%) gambling behavior to a certain extent (*RQ 1a*).

The ordinal logistic regression model including predictors of problem gambling was shown to have a significant and good fit (χ^2^ (16) = 744.30, *p* = 0.000). Furthermore, nearly 16% of the variance in problem gambling can be explained by the variance in the included factors (Nagelkerke R^2^ = 0.157). Table 3 offers an overview of the regression coefficients of all factors. As shown, male respondents (β = 1.21, SE = 0.22, Wald = 30.18, 95% CI = [0.78; 1.64], *p* < 0.00), respondents in the age category 26–35 years old (β = 0.87, SE = 0.44, Wald = 3.93, 95% CI = [0.01; 1.73], *p* < 0.05), and respondents involved in football (β = 0.91, SE = 0.19, Wald = 22.74, 95% CI = [0.54; 1.28], *p* < 0.00) were more likely to end up in a higher category of problem gambling (*RQ 1b*).

### 4.3. The Normalization of Gambling (RQ 2)

Results on the normalization of gambling are presented in three separate subsections, respectively on its (a) availability and accessibility, (b) prevalence and recent and regular implementation, (c) socio-cultural accommodation, and (d) gambling policy attitudes [19]. Nevertheless, most aspects of that second subsection were already discussed above (also see Table 2).

#### 4.3.1. Availability and Accessibility

The availability and accessibility of gambling in sports clubs was assessed by means of measuring whether respondents could engage in sports betting in their club and whether their sports club was organizing sport betting activities. A total of 11% of the respondents disclosed that sports betting was available and accessible in their club, while 11.5% of the respondents indicated that their sports clubs was offering sports betting activities (for example, betting on other games in the same or a different competition) themselves.

#### 4.3.2. Prevalence and Recent and Regular Implementation

Next to the prevalence and recent and regular implementation of gambling we discussed above (as illustrated in Table 2), approximately two-thirds (63.5%) of the last-year gamblers also thought that their friends were frequently participating in sports betting. This result accounts for a strong overestimation of the actual prevalence of sports betting in our sample (15.3%).

#### 4.3.3. Social and Cultural Accommodation

Peer pressure to engage in gambling appeared largely absent in Belgian sports clubs, as only 2.9% of the last-year gamblers indicated pressure from sports friends, whereas only 1% of them expressed participation in gambling activities is required to really “belong” (to be accepted) in the club. Despite that social pressure seemed largely absent, sports betting in particular appeared to be a “part of the game” that was often discussed by last-year gamblers in a positive way with sports friends (63.5%) and coaches (26.9%). Moreover, implying a certain social norm, 46.1% of the last-year gamblers declared that they agree to a certain extent with the statement that their sports friends approve participating in sports betting. Finally, suggesting strong self-perceived behavioral control, 42.3% of the last-year gamblers assumed that financial losses can be limited when one gambles according to a sophisticated system, while 61.5% of them indicated to some extent that winning in sports betting depends on one’s knowledge of sports.

#### 4.3.4. Club Gambling Policy Measures

A vast majority of last-year gamblers (83%) lacked knowledge on where to go when experiencing gambling-related questions and issues, whereas only 2 of them (0.9%) had actively sought help over the past 12 months to deal with their gambling behaviors. Regarding the total study sample, nearly all of the respondents declared that formal rules (96%) and education initiatives (98.7%) on gambling were absent in their sports club. Disclosed initiatives point to one shot actions instead of well-developed policies, illustrated by such examples as their club’s engagement in a seminar on gambling (*n* = 1), a PowerPoint presentation on gambling (*n* = 1), a baseline on ethics and sports (*n* = 1), and this actual survey (*n* = 1). However, in terms of desired gambling policies, respondents showed support for a number of education initiatives (for example, information sessions on gambling risks and regulations and developing an ethical code).

## 5. Discussion

The aim of this study was to examine gambling behaviors and perceptions in sports club actors, using the lens of normalization theory. As implied by participating athletes, coaches, board members, and volunteers, gambling appears to be “part of the game” in Belgian sports clubs. Two research questions were addressed, focusing on the prevalence and predictors of problem gambling as well as on the extent in which gambling is normalized in sports clubs. First, part of our sample of sports club actors indicated low risk (5.7%), moderate risk (4.2%) and problematic (0.8%) gambling behavior (*RQ 1a*). Being a male, being aged 26–35 years old, and being involved in football were exposed as variables that are positively related with higher degrees of problem gambling (*RQ 1b*). Second, the normalization of gambling in Belgian sports clubs was demonstrated by several findings (*RQ 2*). For example, respondents reported a strong engagement in a broad number of gambling activities. Furthermore, the normalization of gambling was also implied by the availability and accessibility of sports betting in sports clubs, by its recent and regular implementation, and by frequent peer discussions and positive socio-cultural norms. Finally, although most respondents declared that gambling rules (96%) and education initiatives (98.7%) were absent in their sports club, they showed support for the development of gambling policies focusing on risks and harms.

Those results that demonstrate the role of age and gender are in line with existing gambling studies, which have explained that young adults and males are more inclined towards (and tempted by) the risk-taking that is inherent to gambling [30]. Additionally, this gender difference might also be influenced by dominant gambling marketing strategies that emphasize gambling as a pre-eminently masculine activity [14]. Moreover, the result in relation to football is empirically novel and could relate to the fact that no sports is as intertwined with gambling as football [13]. Gambling is deeply embedded in many aspects of both playing (for example, gambling companies as shirt sponsors) and consuming football (for example, gambling ads that are linked to football games and talk shows). Next to other socio-cultural links, “football is an ideal vehicle for gambling marketing” [6] (p. 167).

This study further demonstrates that gambling appears to be normalized to a certain extent in Belgian sport clubs. Results on the different components of gambling normalization in sports concord with previous scholarly contributions. For example, respondents overestimated the prevalence of gambling among their sports friends. Such overestimation suggests the presence of a positive socio-cultural norm towards gambling in sports [19]. Hence, it is no surprise that a meaningful proportion of respondents expressed that they discussed gambling (and sports betting in particular) regularly with sports friends and (to a lesser extent) with coaches. Nonetheless, these peer discussions on gambling were not supported by a profound knowledge of the risks and harms of gambling. This result is in accordance with studies in Sweden and Australia that alert for young men and athletes being careless when speaking about gambling [14,18].

Two of the three main drivers of the normalization of gambling were evinced in our study: gambling is socially accepted and positively discussed in Belgian sports clubs [18]. Whereas Deans and colleagues [18] also indicate a strong impact of social pressure, our respondents did not at all articulate they feel pushed to participate in gambling activities by peers or their coaches. However, enforced by gambling’s omnipresence in sports, more subtle and indirect forms of pressure (for example, gambling marketing campaigns) may play a role, of which respondents are largely unaware [11].

### 5.1. Theoretical Contribution

In light of the above, this present study extends and enriches current scientific knowledge on gambling practices and the normalization of gambling in sports in a number of ways. First, applying a view that includes but also goes beyond (elite) athletes, is a direct response to several calls to integrate the perspectives of non-elite athletes and other sports actors such as board members and coaches [14,15,19]. Second, following the suggestion of Derevensky et al. [27] and Vinberg et al. [14], this study exposes certain potential predictors of problem gambling that could be used to prevent gambling-related harms in sports. Third, this study also helps filling in an important void in sports management research, by examining (a) perceptions towards club level gambling policies and (b) socialization mechanisms in sports clubs that contribute to the normalization of gambling [14,15].

Despite the influence of the normalization of gambling on sports (and vice versa), sports management has only paid scarce scholarly attention to how gambling affects different aspects of sports organizations’ operation, including sponsorships, strategic management, and human resources [47]. Accordingly, this study contributes to the development of a new research space in sports management that aims to examine the regulations, learning strategies, and policies implemented by sports organizations to tackle gambling issues. Identifying these elements is necessary to better understand the gambling normalization processes that are currently ongoing in sports.

### 5.2. Practical Implications

When it comes to its practical implications, this study echoes McGee’s [11] (p. 92) suggestion that “greater accountability should also be asked of key stakeholders within sport, including clubs”. While nearly all of the respondents revealed a lack of policies on gambling in their club, there was a positive tendency towards educational and regulatory initiatives on gambling. In fact, these results underscore that ethical leadership and an integrated form of integrity management within sports clubs are paramount to prevent gambling-related harms as much as possible and to effectively deal with these harms when they occur [11]. 

An ethical code outlining organizational values, regulations, and desired behaviors constitutes the first step and the foundation of integrity management in sports clubs [48]. Afterwards, specific educational initiatives, such as developing sessions or workshops on gambling risks and harms, may be implemented to enhance people’s knowledge and awareness of gambling [1,3,19]. Whereas this study’s respondents endorsed the value of such initiatives, their implementation and effectiveness should be evaluated regularly to avoid their misuse as a form of window-dressing, lip service, or “tick the box” mentality [48]. Within these educational initiatives, information on where people can find assistance whenever they experience questions or issues in relation to gambling should be included [44]. Finally, sports clubs should be conscious about the impact of collaborating with the gambling industry and critically assess the visibility of gambling in their club, which both contribute to the normalization of gambling [11].

### 5.3. Limitations and Future Research

When interpreting this study’s results, it is important to note that gambling scholars should be conscious and careful about certain hidden agendas when reporting on problem gambling. While exposing predictors of problem gambling is crucial to prevent gambling-related harms such as financial debts and depression [30], the gambling industry might use their own initiatives to deal with problem gambling on the one hand as a rationale to further legitimize what they call “responsible gambling” on the other hand [30,40]. In a certain sense, forthcoming gambling scholarship should therefore try to shift the attention more from individuals experiencing gambling disorder to problem gambling products (the most addictive products, such as machine slots) [19,44]. Moreover, exclusively focusing on problem gambling might neglect how broadly gambling is embedded and institutionalized from a macro (systemic) viewpoint [12]. Such narrow focus would be unfortunate and even dangerous from a public health perspective, as the normalization of gambling might lead to certain harms for the whole of society [2]. In other words, “gambling behaviour itself does not need to be problematic to cause harm” ([49] (p. 37), as cited in [12] (p. 13)).

This explorative study aims to set the stage for an emerging research agenda on sports organizations’ mediating and moderating roles when it comes to gambling behaviors. Our work offers quantitative insights that must be supplemented with future qualitative and mixed methods work, in line with Foucault’s [31,32] viewpoints on the normalization of social practices. After all, our study is limited in terms of its sample (a relatively small sample compared to the population, including Belgian and sports club data only) and study design (a cross-sectional online survey). Therefore, generalizing and causal claims should be avoided. 

Additionally, the self-selection bias that is inherent to our study design might lead to an overrepresentation of certain demographic groups (for example, males, gamblers), which may distort our results and hinder a perfect foundation for comparisons with the population. This limitation is further implied by the inability to assess the exact response rate of this study due to the chosen administration procedures. Hence, forthcoming studies on gambling that aim to compare prevalence figures in sports with those of the population should ponder the use of total random sampling and weighting techniques. Such an approach would help to determine whether gambling prevalence rates in sports actors are really higher than in the population, which is for instance suggested when cautiously comparing our results on sports betting (5.5 times more prevalent) with the Belgian population figures of Sciensano [46]. Moreover, comparing our data with data collected after the COVID-19 pandemic would help to evaluate potential changes in gambling attitudes and behaviors.

Additionally, as our data were strictly anonymous, more fine-grained multi-level analyses are required to fully exploit how sports clubs (can) contribute to the normalization or denormalization of gambling in sports. Qualitative research (for example, focus groups, participatory observation, or ethnographic work) would support analyses that further improve our understanding about how the socio-cultural environment in sports clubs socializes people into perceiving gambling as fun, harmless, and normal [11]. Existing research also advocates to look into such (often unintended) normalization mechanisms on a meso (organizational or group) level [17,37].

Furthermore, next to personal and socio-cultural influences, we advocate forthcoming studies to examine equally important political, legal, and commercial normalization mechanisms [19]. For example, different sources encourage scholars to engage in research on the impact of gambling sponsorships and advertisement in professional sports clubs on the normalization of gambling [6,18,20]. Building on—and integrating–normalization and institutional theory could provide a richer understanding of the dominant norms and logics underpinning gambling’s firm place in sports and society [17].

Future work is also encouraged to explain differences in (problem) gambling between sports (for example, team sports vs. individual sports) and between sports club actors in a wide range of distinct cultures [11,15]. After all, the current geographical scope of gambling research in sports is quite narrow. Therefore, integrating African and Asian perspectives would enable cross-cultural comparisons of gambling normalization processes. Moreover, given the increasingly blurring lines between sports, eSports, and gaming and gambling, more research on their overlap would support conceptual clarity. Finally, this study encourages scholars to empirically assess how the normalization of gambling creates opportunities to engage in corruption (for example, money laundering and match-fixing), which endangers the integrity of sports and those involved [8,14].

## 6. Conclusions

Drawing on normalization theory and the perceptions of 817 Belgian sports club actors, this study sheds new empirical light on gambling attitudes and behaviors in sports. As such, this study helps to explore the prevalence and normalization of (problem) gambling in sports settings. While doing so, this study’s results also aid to inform gambling policies in sports organizations—and hence—to support the denormalization of gambling in society.

## Figures and Tables

**Table 1:** Sociodemographic characteristics of the study’s sample (*n* = 817).

Variable	Categories	Number	Percentage
Gender	Male	528	64.6
	Female	287	35.1
	X/Transgender	2	0.2
Age category	16–25	315	38.6
	26–35	173	21.2
	36–45	109	13.3
	46–55	93	11.4
	56–65	82	10.0
	65+	45	5.5
Type of sports (top 10)	Football (soccer)	217	26.6
	Tennis	99	12.1
	Volleyball	79	9.7
	Cycling	56	6.9
	Basketball	47	5.8
	Hockey	43	5.3
	Running	43	5.3
	Athletics	35	4.3
	Swimming	34	4.2
	Triathlon	33	4.0
Function within sports club	Athlete	636	77.8
	Board member	193	23.6
	Coaching staff	148	18.1
	Volunteer	100	12.2
	Non-sporting member	27	3.3
Competition level	(Semi)professional	156	19.1
	Amateur/recreational	513	62.8
	No competition	136	16.6

Note. Respondents could select more than one sports club function.

**Table 2:** Last year gamblers involvement in different gambling practices (*n* = 218).

Type of Gambling Activity	Never (%)	Not in the Last Year (%)	<1× a Month (%)	≥1× a Month (%)	≥1× a Week (%)
Lotteries	25.2	8.3	29.8	17.9	18.8
Scratch cards	38.5	15.6	37.2	7.3	1.4
Bingo	82.6	13.8	2.3	0.9	0.5
Poker	65.1	13.8	17.0	4.1	0.0
Machine slots	72.5	14.7	11.5	0.5	0.5
Casino games	65.1	13.3	17.0	4.1	0.5
Sports betting	41.3	7.8	18.3	19.7	10.6
Horse races	88.5	7.8	2.3	0.5	0.9
Other	81.2	12.4	5.0	0.9	0.5

**Table 3:** Output ordinal logistic regression analysis of risk factors for problem gambling in sport (*n* = 817).

Variable	Estimate	Std. Err.	Wald	Sig. (*p*)	95% Confidence Interval
Gender					
Female	−1.21	0.22	30.18	0.00	[−1.64; −0.78]
Male (ref.)					
Age category					
16–25	0.33	0.45	0.56	0.46	[−0.54; 1.20]
26–35	0.87	0.44	3.93	0.05	[0.01; 1.73]
36–45	0.13	0.45	0.08	0.78	[−0.76; 1.02]
46–55	−0.04	0.46	0.01	0.93	[−0.94; 0.87]
56–65	0.19	0.45	0.17	0.68	[−0.70; 1.08]
65+ (ref)					
Level of play					
Non-professional	0.15	0.22	0.47	0.50	[−0.28; 0.58]
(Semi-)professional (ref.)					
Athlete					
No	0.19	0.25	0.56	0.45	[−0.31; 0.68]
Yes (ref.)					
Coach					
No	0.16	0.23	0.50	0.48	[−0.29; 0.61]
Yes (ref.)					
Board member					
No	0.01	0.23	0.00	0.95	[−0.43; 0.46]
Yes (ref.)					
Volunteer					
No	0.39	0.29	1.74	0.19	[−0.19; 0.96]
Yes (ref.)					
Involved in football					
No	−0.91	0.19	22.74	0.00	[−1.28; −0.54]
Yes (ref.)					
Involved in tennis					
No	−0.43	0.24	3.22	0.07	[−0.91; 0.04]
Yes (ref.)					
Involved in basketball					
No	0.61	0.44	1.91	0.17	[−0.26; 1.47]
Yes (ref.)					
Involved in volleyball					
No	−0.52	0.28	3.41	0.07	[−1.07; 0.03]
Yes (ref.)					
Involved in cycling					
No	0.59	0.40	2.16	0.14	[−0.20; 1.37]
Yes (ref.)					

Note: Dependent variable: problem gambling based on the PGSI, including five ordinal categories: non-gamblers, non-problematic gamblers, low risk gamblers, moderate risk gamblers, and problematic gamblers.

## Data Availability

The survey that we used in this study can be downloaded via the following weblink: https://biblio.ugent.be/publication/8754410. The data associated with this study are not publicly available as participants explicitly consented to only have their data shared with the authors.
